# Transfer of the Dominant Virus Resistance Gene *AV-1*^*pro*^ From *Asparagus prostratus* to Chromosome 2 of Garden Asparagus *A*. *officinalis* L.

**DOI:** 10.3389/fpls.2021.809069

**Published:** 2022-02-18

**Authors:** Thomas Nothnagel, Janine König, Jens Keilwagen, Eva-Maria Graner, Jörg Plieske, Holger Budahn

**Affiliations:** ^1^Institute for Breeding Research on Horticultural Crops, Federal Research Centre for Cultivated Plants, Julius Kühn-Institut, Quedlinburg, Germany; ^2^Institute for Biosafety in Plant Biotechnology, Federal Research Centre for Cultivated Plants, Julius Kühn-Institut, Quedlinburg, Germany; ^3^SGS INSTITUT FRESENIUS GmbH, TraitGenetics Section, Gatersleben, Germany

**Keywords:** Asparagus virus 1, resistance, introgression, SNP genotyping array, GBS, pollen

## Abstract

An introgression breeding programme was carried out to transfer the virus resistance gene *AV-1*^*pro*^ from the wild species *Asparagus prostratus* to the garden asparagus *Asparagus officinalis*. Serious crossing barriers caused by genetic distance and different ploidy levels of the crossing parents have been overcome using embryo rescue for the F_1_, BC_1_, and BC_2_ generations. The male and female fertility was widely restored in BC_2_ and was shown to be comparable to the cultivated asparagus. Five AV-1 resistant diploid (2n = 2x = 20) BC_2_ plants were selected and reciprocally backcrossed with asparagus cultivars. Segregation analyses of fourteen seedborne BC_3_ progenies suggested a monogenic dominant inheritance of the AV-1 resistance. Genotyping by sequencing analysis gave a strong hint for location of the resistance gene on asparagus Chromosome 2. Using an Axiom single nucleotide polymorphism (SNP) genotyping array for the analysis of three BC_3_ families with 10 AV-1 resistant and 10 AV-1 susceptible plants each, as well as 25 asparagus cultivars, the *AV-1*^*pro*^ locus on Chromosome 2 was further narrowed down. The SNP with the highest LOD score was converted to a kompetitive allele specific PCR (KASP) marker, shown to be useful for the further backcross programme and serving as the starting point for the development of a diagnostic marker.

## Key Message

Resistance to Asparagus virus 1 was transferred from an asparagus wild relative to garden asparagus by interspecific hybridisation and recurrent backcrosses. The monogenic-inherited *AV-1*^*pro*^ gene is located on Chromosome 2.

## Introduction

Asparagus (*Asparagus officinalis*) is a perennial plant of the Liliaceae family and one of the economically most important vegetable species. The worldwide asparagus production reached 9.43 Mio. tons in 2019 (Statistica, 2021). Until now, nine virus species belonging to the genera *Ilarvirus*, *Cucumovirus*, *Nepovirus*, *Tobamovirus*, *Potexvirus*, and *Potyvirus* have been identified in asparagus (Tomassoli et al., 2012). Among these, the potyvirus *Asparagus virus 1* (AV-1), a filamentous virus measuring 700–880 nm in length and 13 nm in width (Fujisawa et al., 1983), is the most important virus attacking garden asparagus worldwide. While the damage caused by AV-1 is often underestimated, because no symptoms are visible on shoots or cladophylls, various studies reported yield losses between 30 and 70% (Weissenfels and Schmelzer, 1976; Yang, 1979; Evans et al., 1990; De Vries-Paterson et al., 1992). A more recent greenhouse study by Lantos et al. (2018) has demonstrated that AV-1 infection restricts the root development (root weight, ratio of storage root, and number of spear meristems), reduces the Brix value, and influences the quantity of terpenoids and other metabolic compounds.

The influence of virus infection on spear quality and susceptibility to other pathogens such as *Fusarium* spp. was discussed intensively (Yang, 1979; Greiner, 1980; Fujisawa et al., 1983; Evans and Stephens, 1984; Evans et al., 1990; Elmer et al., 1996). It was estimated that European asparagus plantations had an infestation degree of 90–100% in the last decades (Kegler et al., 1999; Bandte et al., 2008; Knaflewski et al., 2008; Tomassoli et al., 2008; Nothnagel et al., 2013). The main reason is the easy virus transmission by aphids and mechanical transmission, e.g., during harvest. Furthermore, rapid spreading of AV-1 is supported by a high regional concentration of the production areas and the immediate replanting of asparagus fields (reduced or no crop rotation) as a result of limited suitable acreage (Hein, 1963; Conroy, 1975; Weissenfels and Schmelzer, 1976; Young, 1984).

Comprehensive studies have shown that there are no AV-1 resistant cultivars of garden asparagus available (Howell, 1985; Kegler et al., 1999; Knaflewski et al., 2008). Evaluation of 44 asparagus cultivars and 31 wild relatives of asparagus failed to detect resistance to AV-1 in any of the cultivars, while 20 of the 31 wild relatives were resistant (Nothnagel et al., 2017). Until now, there has been no information about the genetic background of the different resistance sources. However, vector resistance was excluded for all these resistance sources by testing the feeding behaviour of the aphid *Myzus persicae* using an electrical penetration graph (EPG) technique (Lantos et al., 2019).

An introgression breeding programme was initiated to transmit putative AV-1 resistance genes from asparagus wild species of different ploidy levels to the garden asparagus (Plath et al., 2018). This article describes the development of AV-1-resistant diploid garden asparagus breeding lines from the interspecific cross between *A. officinalis* and tetraploid *Asparagus prostratus*, the study of the inheritance mode of the resistance gene *AV-1*^*pro*^, and the identification of a useful kompetitive allele specific PCR (KASP) marker for application in breeding and breeding research.

## Materials and Methods

### Plant Material, Crosses, Segregation Analysis

**FIGURE 1:**
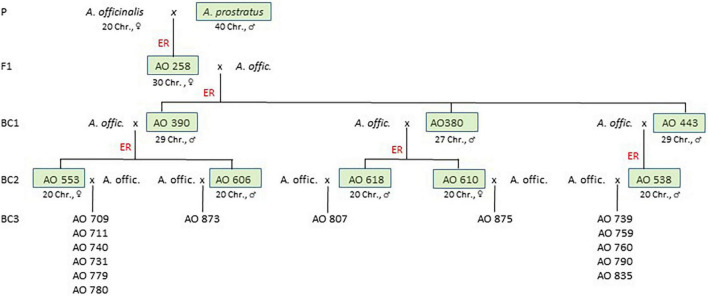
Simplified pedigree of the introgression programme based on an interspecific cross between *A. officinalis* and *A. prostratus* reduced to relevant branches described in this article. Each of the *A. officinalis* parents at the cross generations of F_1_ up to BC_2_ generation represents a plant of an individual cultivar (compare Supplementary Tables 1, 2). The *A. officinalis* parents of the BC_3_ progenies represent plants of various cultivars listed in Table 1 and Supplementary Table 1. The BC_3_ progenies are seed-borne segregation populations analysed in this genetic study (boxes marked the AV-1-resistant cross parents, ER, embryo rescue; Chr, chromosomes).

Seed material of asparagus cultivars (*A. officinalis* L.) and the wild relative *A. prostratus* Dumort. was obtained from various breeders and genebanks (Supplementary Table 1). A section of a complex backcross programme relevant to the genetic analysis of the AV-1^*pro*^ resistance is shown in Figure 1. For the initial interspecific cross (F_1_), the first (BC_1_), and second backcrosses (BC_2_) embryo rescue were necessary (described by Plath et al., 2018).

**TABLE 1:** Results of the BC_3_ crossing experiment, the resistance test, and the segregation analysis.

No. BC3	Cross combination* Female × Male	Flowers pollinated	Fruits set		Seeds harvested	Seeds per fruit	Sowing	Germinated seeds	Germination in %	AV-1		Chi2 (1:1)
			No	%						Resistant	Susceptible	
	*BC_2_ × A. officinalis*											
AO 709	AO 553 × SWM	17	16	94.12	44	2.75	42	34	80.95	17	17	1.000
AO 711	AO 553 × BOO	10	6	60.00	9	1.50	9	9	100.00	7	2	0.096
AO 731	AO 553 × EPO	16	8	50.00	15	1.88	15	13	86.67	7	6	0.782
AO 740	AO 553 × RAV	17	10	58.82	29	2.90	28	18	64.28	6	12	0.157
AO 779	AO 553 × DAR	29	20	68.97	46	2.30	46	39	84.78	18	21	0.631
AO 780	AO 553 × DOR	7	7	100.00	22	3.14	22	12	54.55	6	6	1.000
AO 875	AO 610 × RAV	5	4	80.00	11	2.75	11	7	63.64	5	2	0.257
Total – male parent *A. offic.*		101	71	70.30	176	2.49	173	132	76.30	66	66	1.000
	*A. officinalis* × *BC*_2_											
AO 739	BL1 × AO 538	59	16	27.12	22	1.38	21	15	71.43	6	9	0.438
AO 759	SWM × AO 538	28	9	32.14	35	3.89	35	34	97.14	15	19	0.493
AO 760	BL2 × AO 538	10	6	60.00	10	1.67	9	8	88.89	4	4	1.000
AO 790	SWM × AO 538	38	21	55.26	63	3.00	62	43	69.35	17	24	0.274
AO 807	SWM × AO 618	5	3	60.00	9	3.00	8	7	87.50	3	4	0.705
AO 835	SWM × AO 538	63	52	82.54	193	3.71	60	44	73.33	16	28	0.050
AO 873	SWM × AO 606	46	26	56.52	63	2.42	63	46	73.02	23	23	1.000
Total – female parent *A. offic.*		249	133	53.41	395	2.97	258	197	76.36	84	111	0.053
Total		350	204	58.29	571	2.79	431	329	76.33	150	177	0.135

*A. officinalis parent (cv.): BL1/BL2, breeding lines; BOO, Boonlim; DAR, Darlise; DOR, Dorsiane; EPO, Eposs; RAV, Ravel; SWM, Schwetzinger Meisterschuss.

Selected diploid (2n = 2x = 20) AV-1-resistant BC_2_ plants and seed-borne plants of various asparagus cultivars were cultivated in plastic pots under optimised greenhouse conditions up to flowering (Supplementary Table 2). Reciprocal crosses have manually been performed under insect-protected conditions. After approximately 2 months, BC_3_ seeds were harvested and sown in a sand-humus mixture (3:1 v/v). The seedlings had been cultivated in 9-cm plastic pots under insect-protected greenhouse conditions before resistance screening. Screening results were used for a segregation analysis. A chi-square test was used to test goodness-of-fit to expected ratios for monogenic-dominant inheritance in the BC_3_ generation (1:1) (Table 1).

### Resistance Screening

The resistance screening was performed as a climatic chamber assay following the protocol described by Nothnagel et al. (2017). The green peach aphid *Myzus persicae* vector has continuously been kept virus free in acrylic glass cages with gauze sides as parthenogenetic population on sweet pepper plants (*Capsicum annuum* L. “Pusztagold”). The test plants were placed in a climate chamber at 22°C D/N, a 16-h-day length at photosynthetic active radiation (PAR) of 10,000 l m/m^2^ and approximately 60% relative humidity. The aphids were placed on AV-1-infested *A. officinalis* donor plants surrounded by test plants to facilitate aphid migration and virus transmission. The migration of the aphids from the donor to the test plants was controlled daily. Seven days later, the aphids were eliminated through spraying with 0.035% Confidor WG 70 (Bayer Crop Science, Langenfeld, Germany).

Six weeks after incubation, the plants were tested for appearance of AV-1 using a DAS-ELISA (Clark and Adams, 1977) with polyclonal antibodies obtained from the JKI serum bank (H. Ziebell). The absorbance was measured using a microplate-reader TECAN^®^ Sunrise (Männedorf, Switzerland) with the analysis software Magellan 7.2 SP1 STD2PC at 405 nm. All plants that were tested negative for AV-1 (ELISA < 0.1) underwent second and third rounds of resistance screening. Those that were confirmed triple negative were declared to be AV-1 resistant.

### Chromosome Analysis

One-centimeter-long root tips were cut from pot-grown plants and collected in distilled water. They were placed on ice for 1–1.5 h and treated with 2-mM 8-hydroxychinoline for 2.5 h. Afterward, root tips were fixed in freshly prepared ethanol acetic acid (3:1) for at least 24 h at room temperature and stored at 4°C until analysis. After a 5-min-washing step with distilled water, the root tips were digested with an enzyme mixture of 4% cellulase (“Onozuka R-10,” Duchefa Biochemie, Haarlem, Netherlands) and 1% pectolyase Y-23 (Seishin Pharmaceutical Co., Tokyo, Japan) in 75-mM KCl and 7.5-mM Na_2_-EDTA solution (pH 4.0) for 45 min at 37°C (Kakeda et al., 1991). The macerated meristem was washed after removal of the enzyme mixture for 10 min in distilled water and squashed in 45% acetic acid. The chromosomes were counted using the phase contrast of an Axioskop 2 microscope (Zeiss, Oberkochen, Germany).

### Analysis of Pollen Viability

The pollen viability was determined by vital staining of microspore cells, using fluorescein diacetate (FDA) according to Heslop-Harrison and Heslop-Harrison (1970). From each tested male plant, at least four juvenile flower buds were collected, and one anther per flower was immediately suspended in 0.5 ml of a solution containing 75-mg/ml sucrose and 1-mg/ml FDA (Serva Electrophoresis GmbH, Heidelberg, Germany). Pollen viability was determined as a relative ratio of pollen emitting strong and uniform fluorescence (highly vital) when subjected to UV light using a Nikon 90i microscope (Nikon, Düsseldorf, Germany), equipped with a fluorescence filter set MBE 41300 (EX340-380/BA435-485 nm). Pollen grains with abnormal shape, without or with weak fluorescence, were classified as with low vitality or dead. At least 800 pollen grains per anther were investigated.

### DNA Isolation and Genotyping by Sequencing (GBS)

Total genomic DNA was isolated from 100 mg of young cladophyll tissue using the innuPREP Plant DNA Kit (Analytik Jena, Jena, Germany), quantified by NanoDrop 8000 UV/Vis spectrophotometer (Peqlab, Erlangen, Germany) and sent to LGC Genomics (Berlin, Germany) for library construction and sequencing (150 bp paired-end, Illumina NextSeq). LGC used an optimised, self-tuning GBS method, called normalised GBS (nGBS), which is based on the restriction enzyme *Msl*I to produce blunt end fragments, and an additional enzyme treatment in a subsequent normalisation step. After sequencing, GBS reads were de-multiplexed according to the sample barcodes, and sequencing adapter remnants were removed using a GBS barcode splitter. Reads were trimmed by eliminating low Phred quality bases (Q score <20) and reads with a final length shorter than 20 base pairs before mapping and single nucleotide polymorphism (SNP) calling. The asparagus reference genome and the Aspof.V1 reference annotation release 100 were downloaded from Phytozome (Goodstein et al., 2012), and trimmed GBS reads were mapped against this genome using BWA-MEM (version: 0.7.7.-r1140) (Li, 2013).

Although the accessions had different numbers of chromosomes and ploidy, variant calling was performed assuming diploid genomes for all accessions. Variant calling was performed with samtools (version: 1.2) and bcftools (version: 0.1.19-96b5f2294a) (Li et al., 2009). Subsequently, the raw variants were further filtered (non-monomorphic SNPs; min. SNP quality = 40; min. GT quality = 5; min. number of reads, covering a position per sample = 4; percentage of uncovered samples per SNP = 0.5) to obtain a matrix of 44,805 high-quality bi-allelic SNPs for further analyses.

### Axiom Single Nucleotide Polymorphism (SNP) Genotyping Array Analysis

For SNP genotyping, a recently developed asparagus Axiom SNP genotyping array of the SGS INSTITUT FRESENIUS GmbH, TraitGenetics Section (Gatersleben, Germany) was used. A detailed description of this array is currently in preparation. In brief, this array has been developed based on SNPs identified from the re-sequencing of six asparagus varieties and subsequent SNP identification in these lines through a comparison to the reference sequence (Aspof.V1 reference annotation release 100). From an initial set of 165,106 SNP markers that fulfilled assay design criteria, 41,953 SNP markers were selected for an Axiom array (Thermo Fisher Scientific Inc., Waltham, MA, United States). After initial screening of the array with a set of asparagus lines, approximately 24,000 high-quality asparagus SNP markers remained. The array was termed AxAO024 and was used for scoring in the investigated material of this study using the Axiom Analysis Suite Software.

### Statistics

To identify the genetic region that harbours the resistance gene, case control studies were performed using an SNP matrix in Variant Calling Format (VCF) either from GBS or from the AxAO024 array. These analyses were performed with SnpSift 4.0 (Cingolani et al., 2012) using a simple TFAM file that encodes each accession in a separate line. Resistant and susceptible accessions differ in the value of Column 6, which is 1 for case and 2 for control, respectively. Assuming a monogenic-dominant resistance, *p*-values were extracted from CC_DOM, and LOD values were computed. Chromosomal and positional information was used in combination with the LOD values to create Manhattan plots (R Core Team, 2020).

### Kompetitive Allele Specific PCR – Marker

Sequence information from 200 bp upstream and downstream of the SNP Ax-553065352 from the AxAO024 array was provided to LGC Genomics (Berlin, Germany) for KASP assay development. About 5-μl samples containing 50 ng of genomic plant DNA, 2.5-μl LGC universal KASP-TF master mix, and 0.07 μl of the specific primer mix (fw-primer 1: 5′-TCAAATAATAAGTAAATGTGGTTTATTTGCTC-3′; fw-primer 2: 5′-CAAATAATAAGTAAATGTGGTTTATTTGCTG-3′; rev-primer: 5′-GACGGGGTTTTCACAGGTACACAAA-3′) were analysed using the following PCR protocol: 15 min at 94°C, 10 cycles with 20 s at 94°C and 60 s at 61°C with a decrement of 0.6 grd/s, followed by 26 cycles with 20 s at 94°C and 60 s at 55°C and finalised for 60 s at 37°C. If necessary, recycling (three cycles) has been performed with 20 s at 94°C and 60 s at 57°C, and 60 s at 37°C before measurement. Allelic discrimination has automatically been performed using CFX Maestro Software (Bio-Rad, Munich, Germany).

## Results

### Backcross Programme *A. officinalis* × *A. prostratus*

The introduction and comprehensive data to the whole introgression crossing programme with various wild relatives from the initial cross (F_1_) up to BC_2_ were published by Plath et al. (2018). The following chapter describes the backcrossing approach relevant to the introgression of the AV-1 resistance from *A. prostratus* and its genetic analysis.

Crossing of diploid *A. officinalis* (2n = 2x = 20) with tetraploid *A. prostratus* (2n = 4x = 40) was successful. Twenty-nine of 80 crossed flowers (36%) developed berries, from which 17 embryos were prepared and 14 were finally rescued *in vitro*. Established *in vitro* plants of six embryos were cloned, and 104 F_1_ hybrid plants were transferred into soil. Twenty-seven F_1_ hybrid plants were determined as resistant. Resistant F_1_ plants with 30 chromosomes were backcrossed (BC_1_) with various *A. officinalis* cultivars. About 625 crosses resulted in sixty juvenile berries, but only 19 embryos were rescued *in vitro* and cloned. From 62 established BC_1_ plants, 21 showed resistance to AV-1. The chromosome number varied between 24 and 30.

About 388 crosses between AV-1-resistant BC_1_ plants and *A. officinalis* resulted in 77 berries. In total, 54 embryos were prepared, from which 39 primary BC_2_ plants were regenerated. After cloning and transfer into soil, 289 BC_2_ plants were tested for AV-1. In total, 147 plants of 16 crossing events were resistant (Supplementary Table 2 and Figure 1). For five AV-1-resistant BC_2_ plants, a reduction of the chromosome number to the diploid level (2n = 2x = 20) was shown (Supplementary Figure 1). The two diploid female BC_2_ clones AO 553 and AO 610 and three male clones AO 538, AO 606, and AO 618 were backcrossed (BC_3_) with different asparagus cultivars (Figure 1 and Table 1).

Altogether, 350 BC_3_ crosses were made, whereof 204 set a berry (58.3%) with approximately 2.79 seeds per berry. There was a tendency for fruit set to be lower in the crosses with BC_2_ as the male parent (pollinator, 53.4 vs. 70.3%), while seed set per berry and seed germination were comparable in the reciprocal crosses. From the 431 sowed seeds, 76.3% germinated, and 14 crossing progenies (BC_3_) were established with 7–46 individual plants (Table 1 and Figure 1). All of the 14 tested BC_3_ progenies segregated into resistant and susceptible plants in a ratio fitting to a Mendelian monogenic-dominant inheritance (1:1).

### Plant Habit

As the name suggests, *A. prostratus* grew as a prostrate plant with 20- to 40-cm recumbent stems, whereas the *A. officinalis* developed long erect stems up to two-metre high. The F_1_ plants showed a habit more similar to the *A. prostratus* parent with prostrate or ascending obliquely stems. The phenotype of the BC_1_ varied between nearly erect, ascending obliquely, and drooping. All BC_2_ and BC_3_ plants expressed a habit highly similar to the *A. officinalis* parent but slightly compressed (Supplementary Figures 2, 3). All cross generations segregated into female and male plants (Supplementary Table 1 and Figure 1). The female and male flower architecture (Supplementary Figure 4) and berry development were expressed in highly similar manner, in both cross parents and all crossing progenies.

### Pollen Fertility

The pollen fertility of the wild relative *A. prostratus* and the *A. officinalis* cultivars used as pollinators varied between 73 and 81%. As expected, increasing pollen fertility was observed at higher backcross generations and for reduced chromosome numbers. Whereas, in anthers of male F_1_ plants (30 Chr.), only a low frequency of fertile pollen (<10%) was detected; BC_1_ plants reached up to 54% pollen fertility. A more or less completely restored pollen fertility was observed for the BC_2_ plants; only AO 618 showed significant lower pollen fertility in comparison to the tested *A. officinalis* cultivars (Supplementary Table 3 and Supplementary Figure 5).

### GBS Analysis

**FIGURE 2:**
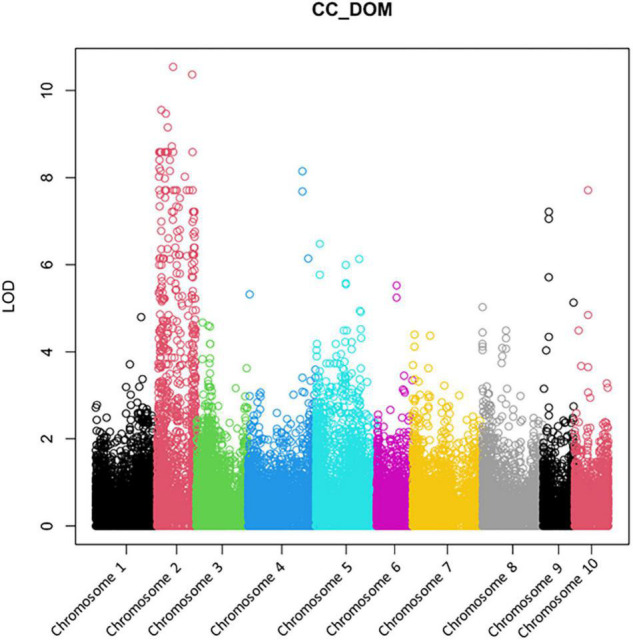
A Manhattan plot of the GBS analysis using data of 32 AV-1-susceptible and 19 AV-1-resistant plants from the pedigree of the backcross programme and cultivars (Supplementary Table 4). The genome comparison shows a clear association of the AV-1 resistance with asparagus Chromosome 2.

For GBS analysis and subsequent case control studies, 32 AV-1 susceptible and 19 AV-1-resistant plants from the pedigree of the backcross programme (from BC_1_ to BC_2_ generations) and cultivars (Supplementary Table 4) were used. Figure 2 demonstrates a clear association of AV-1 resistance with asparagus Chromosome 2. Nevertheless, the undefined distribution of high LOD scores over the complete Chromosome 2 did not permit the exact localisation of the resistance gene.

### Array Analysis

Other than for GBS analysis, for the array already, BC_3_ progenies were available. About 92 DNA samples (Supplementary Table 5) were analysed with the AxAO024 array. About 24,189 asparagus SNPs were polymorphic in the set. The number of markers per chromosome ranged from 3,687 for Chromosome 4 and 1,023 for Chromosome 9.

**FIGURE 3:**
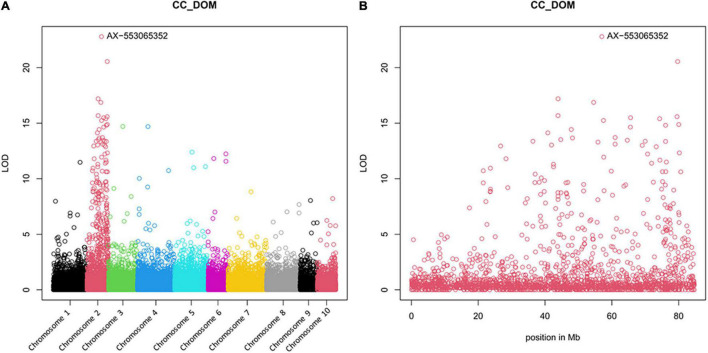
Manhattan plots of the array analysis, resulting from 30 diploid AV-1-resistant BC_3_ plants, 30 diploid AV-1-susceptible BC_3_ plants, the resistant and susceptible parental plants, and 25 AV-1-susceptible cultivars. **(A)** For all 10 asparagus chromosomes; **(B)** Specifically for asparagus Chromosome 2. The genome comparison showed an association of AV-1 resistance with marker Ax-553065352 on asparagus Chromosome 2.

The Manhattan plot confirms the localisation of the resistance gene on Chromosome 2 as predicted by GBS analysis (Figure 3). For the marker with the highest LOD score, AX-553065352, a nearly perfect accordance of genotype and resistance behaviour of the individual plants, has been shown (Supplementary Table 6).

### kompetitive Allele Specific PCR Marker

**FIGURE 4:**
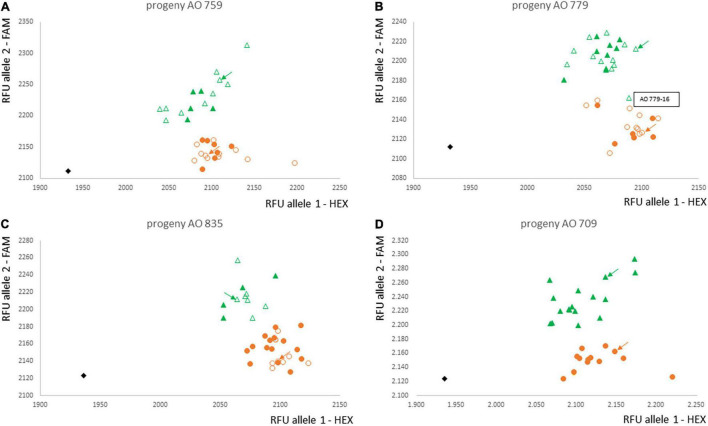
Validation of marker KASP-Ax-553065352 for the complete progenies AO 759 **(A)**, AO 779 **(B)**, AO 835 **(C)**, and the progeny AO 709 not used for the array **(D)**. Orange circles: homozygous *A. officinalis* marker allele; green triangle: heterozygous; black rhomb: water control; unfilled symbols: already analysed with array; filled symbols: newly analysed plants; plant AO 779-16: homozygous marker type but AV-1-resistant phenotype; green and orange arrows: parental plants; RFU, relative fluorescence units; HEX and FAM: used fluorescence dyes.

The marker Ax-553065352 was converted into a KASP marker. Validation of the KASP-AX-553065352 marker was performed with the entire progenies AO 759, AO 779, and AO 835 and not only the 20 plants used for the microarray analysis (Figures 4A–C and Supplementary Table 5). The results were completely consistent for the plants analysed with the array and the KASP marker. Only one AV-1-resistant plant, AO 779-16, had the marker genotype in the microarray and KASP analysis that was associated with AV-1 susceptibility of all other plants. A fourth round of AV-1 resistance testing was performed to rule out the possibility that the plant was incorrectly characterised as resistant. Even after an additional fourth resistance test, this plant was clearly characterised as resistant. Consequently, this plant can be considered to have a recombination event between the marker locus and the resistance locus.

In addition, progeny AO 709 was analysed with the KASP marker as independent validation, since this progeny was not previously used in any analysis to determine the chromosomal region or the KASP marker. The KASP genotypes fully matched the AV-1-resistance phenotypes. No additional recombination was identified (Figure 4D).

## Discussion

The transfer of a resistance to potyvirus AV-1 from the tetraploid wild relative *A. prostratus* to the cultivated *A. officinalis* was realised by interspecific crosses and an introgression breeding strategy demonstrated by Prohens et al. (2017) to be a probate tool. In general, interspecific crosses in the genus asparagus are considered to be very difficult (Falavigna et al., 2008; Kanno and Yokoyama, 2011). The only publication by McCollum (1988), reporting successful crosses between *A. prostratus* and *A. officinalis*, provides no information about further usage in actual breeding approaches.

Embryo rescue was necessary in F_1_, BC_1_, and BC_2_ generation to overcome crossing barriers, making the project complicated and time-consuming. Because of the tetraploid state of resistance donor *A. prostratus*, several backcrossing steps with *A. officinalis* were necessary. However, chromosome counting in each backcross generation for selection of the plants with the lowest chromosome number allowed the development of diploid AV-1-resistant garden asparagus breeding lines already 8 years after the initial crosses.

In the original wild population of the resistance donor *A. prostratus*, five resistant and 12 susceptible plants were identified (Nothnagel et al., 2017). This argues for genetically determined resistance. Even in the primary cross (F_1_) and the following backcross generation (BC_1_), segregation in resistant and susceptible plants was observed (Plath et al., 2018). In the literature, dominant resistances are mostly involved in active defence reactions such as hypersensitive response (HR). In contrast, the recessive resistances usually correspond to loss or mutations of host factors responsible for the virus life cycle (Robaglia and Caranta, 2006).

Appearance of AV-1-resistant diploid (2n = 2x = 20) BC_2_ plants with nearly normal pollen fertility and seed set in comparison to *A. officinalis* was a precondition to study the genetic inheritance of the AV-1 resistance in various BC_3_ progenies. Because male and female diploid BC_2_ plants were available, recurrent crosses have been done to study effects of the crossing direction. The segregation analysis of the BC_3_ progenies suggested a goodness-of-fit to monogenic-dominant inheritance of the AV-1 resistance. The same segregation pattern in different cross combinations further suggests a stable and potent introgression, which enables the use of the material for commercial breeding programmes.

GBS analysis of resistant and susceptible individual plants of the BC_1_ and BC_2_ generation, as well as susceptible plants from garden asparagus cultivars, was useful for assignment of the resistance to asparagus Chromosome 2. To map the resistance gene more exactly, 10 AV-1-resistant and 10 susceptible plants of three BC_3_ progenies and 25 asparagus cultivars were genotyped using the AxAO024 microarray from SGS. The marker with the highest LOD score, AX-553063352, is located within an unusual large intron of the calnexin (cnx) gene of the *A. officinalis* reference genome. The proteins calnexin and calreticulin (crt) are known as molecular chaperones and fulfil essential functions for the folding of cellular and viral glycoproteins and quality control of the endoplasmatic reticulum (Parodi, 2000). Both proteins play a role in plant perception under stress conditions (reviewed by Garg et al., 2015). It is unclear if the resistance-causing gene can be *cnx* or an adjacent gene because one recombination between marker AX-553063352 and the resistance gene in the tested set of 161 plants (microarray and KASP analyses together; Supplementary Table 5) was found, and recombination frequency between the introduced *A. prostratus* fragment and *A. officinalis* is unknown. However, it is clear that this marker must be quite tightly linked with the actual resistance gene. For supporting the backcross progress in breeding and breeding research, the best marker was converted to a user-friendly KASP-marker. The results with this marker were completely consistent with that of the array. Validation of the marker has been performed with further plants of the progenies used with the array, but also with an independent population.

Until now, no other virus resistance gene has been mapped in asparagus. Nevertheless, potyviruses share many structural and biochemical properties and form a phylogenetically homogeneous group of recent radiation (Gibbs and Ohshima, 2010). It is often assumed that they also engage in essentially similar cellular interactions with their hosts (Ouibrahim et al., 2015). For other potyviruses, like Papaya ringspot virus (PRV), Potato virus Y (PVY), and Lettuce mosaic virus (LMV), remarkable progress has been made to understand the nature of plant resistance to viruses at the molecular level. Common dominant plant defence strategies against viral attacks are R-gene-mediated resistances like TIR-NB-LRR (Vidal et al., 2002; Brotman et al., 2013) and CC-NB-LRR (Hayes et al., 2004). Therefore, the identification of the resistance gene should be an object of further research.

## Data Availability Statement

The datasets presented in this study can be found in online repositories. The sequence reads have been deposited into the ENA (EMBL-EBI) study PRJEB48367.

## Author Contributions

TN: plant development, genetics, and manuscript conception. JKö: AV-1 test approach. JKe: GBS analysis and statistics. HB: molecular markers and manuscript conception. E-MG: SGS IF TG – *Asparagus* Axiom microarray. JP: a KASP marker and Axiom analysis. All authors contributed to the article and approved the submitted version.

## Conflict of Interest

JP and E-MG were employed by SGS INSTITUT FRESENIUS GmbH, TraitGenetics Section. The remaining authors declare that the research was conducted in the absence of any commercial or financial relationship that could be constructed as a potential conflict of interest.

## Publisher’s Note

All claims expressed in this article are solely those of the authors and do not necessarily represent those of their affiliated organizations, or those of the publisher, the editors and the reviewers. Any product that may be evaluated in this article, or claim that may be made by its manufacturer, is not guaranteed or endorsed by the publisher.
