# Supplementary material for: Transfer of the Dominant Virus Resistance Gene AV-1pro From Asparagus prostratus to Chromosome 2 of Garden Asparagus A. officinalis L
Source: Front Plant Sci. 2022 Feb 18;12:809069. doi: 10.3389/fpls.2021.809069 (PMC8895299; doi:10.3389/fpls.2021.809069)
Supplement: Supplementary file 1 [file Data_Sheet_1.PDF]

## **Table S1** Plant material used in the crossing experiments

| No | Species / pedigree | Seed / plant origin | Description[1] | Sex[2] | Ploidy | AV-1[3] |
|---|---|---|---|---|---|---|
| PRO | *Asparagus prostratus* Dumort. | Vilmorin (FRA) - Honat Is., France* | Wild relative | | 2n = 4x = 40 | res/susc |
| | *Asparagus officinalis L.* | | | | | |
| RAV | cv. Ravel | Südwestdeutsche Saatzucht GmbH Rastatt (DEU) | All male $F_1$ hybrid | M | 2n = 2x = 20 | susc |
| DAR | cv. Darlise | Darbonne - Inotalis (FRA) | All male $F_1$ hybrid | M | 2n = 2x = 20 | susc |
| DOR | cv. Dorsiane | Planasa/Inotalis, Valtierra (Spain) | All male $F_1$ hybrid | M | 2n = 2x = 20 | susc |
| SWM | cv. Schwetzinger Meisterschuß | Südwestdeutsche Saatzucht GmbH Rastatt (DEU) | OP-cultivar | F / M | 2n = 2x = 20 | susc |
| GIL | cv. Gijnlim | Limgroup B.V. (NLD) | All male $F_1$ hybrid | M | 2n = 2x = 20 | susc |
| EPO | cv. Eposs | Südwestdeutsche Saatzucht GmbH Rastatt (DEU) | $F_1$ hybrid | F/M | 2n = 2x = 20 | susc |
| BOO | cv. Boonlim | Limgroup B.V. (NLD) | $F_1$ hybrid | F / M | 2n = 2x = 20 | susc |
| BL1 | Breeding line [AO 172] | JKI Collection (Quedlinburg, DEU) | Cross population | F / M | 2n = 2x = 20 | susc |
| BL2 | Breeding line [AO 2] | JKI Collection (Quedlinburg, DEU) | Cross population | F | 2n = 2x = 20 | susc |
| BL3 | Breeding line [AO 3] | JKI Collection (Quedlinburg, DEU) | Cross population | M | 2n = 2x = 20 | susc |
| BL4 | Breeding line [AO 4] | JKI Collection (Quedlinburg, DEU) | Cross population | M | 2n = 2x = 20 | susc |
| | *A.officinalis* x *A.prostratus* hybrids (Generation) | | | | | |
| AO 234 | BL1 x PRO ($F_1$) | JKI - Pre-breeding programme | ER clone | F | 2n = 3x = 30 | res |
| AO 246 | BL1 x PRO ($F_1$) | JKI - Pre-breeding programme | ER clone | F | 2n = 3x = 30 | res |
| AO 250 | BL1 x PRO ($F_1$) | JKI - Pre-breeding programme | ER clone | M | 2n = 3x = 30 | res |
| AO 252 | BL1 x PRO ($F_1$) | JKI - Pre-breeding programme | ER clone | F | 2n = 3x = 30 | res |
| AO 258 | BL1 x PRO ($F_1$) | JKI - Pre-breeding programme | ER clone | F | 2n = 3x = 30 | res |
| AO 297 | BL1 x PRO ($F_1$) | JKI - Pre-breeding programme | ER clone | F / M | 2n = 3x = 30 | res |
| AO 380 | AO 258 x BOO ($BC_1$) | JKI - Pre-breeding programme | ER clone | M | 2n=27 | res |
| AO 390 | AO 258 x BOO ($BC_1$) | JKI - Pre-breeding programme | ER clone | M | 2n=29 | res |
| AO 435 | AO 258 x BOO ($BC_1$) | JKI - Pre-breeding programme | ER clone | F | 2n=28 | res |
| AO 443 | AO 258 x BOO ($BC_1$) | JKI - Pre-breeding programme | ER clone | M | 2n=29 | res |
| AO 449 | AO 258 x BOO ($BC_1$) | JKI - Pre-breeding programme | ER clone | F | 2n=29 | res |
| AO 538 | SWM x AO 443 ($BC_2$) | JKI - Pre-breeding programme | ER clone | M | 2n = 2x = 20 | res |
| AO 553 | BL2 x AO 390 ($BC_2$) | JKI - Pre-breeding programme | ER clone | F | 2n = 2x = 20 | res |
| AO 606 | BL2 x AO 390 ($BC_2$) | JKI - Pre-breeding programme | ER clone | M | 2n = 2x = 20 | res |
| AO 610 | BL2 x AO 380 ($BC_2$) | JKI - Pre-breeding programme | ER clone | F | 2n = 2x = 20 | res |
| AO 618 | BL2 x AO 380 ($BC_2$) | JKI - Pre-breeding programme | ER clone | M | 2n = 2x = 20 | res |
| AO 627 | BL2 x AO 380 ($BC_2$) | JKI - Pre-breeding programme | ER clone | M | 2n = 2x = 20 | susc |
| AO 632 | BL2 x AO 380 ($BC_2$) | JKI - Pre-breeding programme | ER clone | M | 2n = 2x = 20 | susc |
| AO 709 | AO 553 x SWM ($BC_3$) | JKI - Pre-breeding programme | cross seeds | n.d. | 2n = 2x = 20 | res/susc |
| AO 711 | AO 553 x BOO ($BC_3$) | JKI - Pre-breeding programme | cross seeds | n.d. | 2n = 2x = 20 | res/susc |
| AO 731 | AO 553 x EPO ($BC_3$) | JKI - Pre-breeding programme | cross seeds | n.d. | 2n = 2x = 20 | res/susc |
| AO 739 | BL1 x AO 538 ($BC_3$) | JKI - Pre-breeding programme | cross seeds | n.d. | 2n = 2x = 20 | res/susc |
| AO 740 | AO 533 x RAV ($BC_3$) | JKI - Pre-breeding programme | cross seeds | n.d. | 2n = 2x = 20 | res/susc |
| AO 759 | SWM x AO 538 ($BC_3$) | JKI - Pre-breeding programme | cross seeds | n.d. | 2n = 2x = 20 | res/susc |
| AO 760 | BL2 x AO 538 ($BC_3$) | JKI - Pre-breeding programme | cross seeds | n.d. | 2n = 2x = 20 | res/susc |
| AO 779 | AO 553 x DAR ($BC_3$) | JKI - Pre-breeding programme | cross seeds | n.d. | 2n = 2x = 20 | res/susc |
| AO 780 | AO 553 x DOR ($BC_3$) | JKI - Pre-breeding programme | cross seeds | n.d. | 2n = 2x = 20 | res/susc |
| AO 790 | SWM x AO 538 ($BC_3$) | JKI - Pre-breeding programme | cross seeds | n.d. | 2n = 2x = 20 | res/susc |
| AO 807 | SWM x AO 618 ($BC_3$) | JKI - Pre-breeding programme | cross seeds | n.d. | 2n = 2x = 20 | res/susc |
| AO 835 | SWM x AO 538 ($BC_3$) | JKI - Pre-breeding programme | cross seeds | n.d. | 2n = 2x = 20 | res/susc |
| AO 873 | SWM x AO 606 ($BC_3$) | JKI - Pre-breeding programme | cross seeds | n.d. | 2n = 2x = 20 | res/susc |
| AO 875 | AO 610 x RAV ($BC_3$) | JKI - Pre-breeding programme | cross seeds | n.d. | 2n = 2x = 20 | res/susc |

*Seeds of *Asparagus prostratus* was received from G. Simon (Vilmorin, FRA), who collected seeds at the Honat Is., France, in the 1980ies.

[1]OP - open pollinated cultivar, ER - Embryo rescue; [2]F - Female, M - Male, n.d. - not determined so far; [3]res - resistant, susc - susceptible
