# Supplementary material for: Transfer of the Dominant Virus Resistance Gene AV-1pro From Asparagus prostratus to Chromosome 2 of Garden Asparagus A. officinalis L
Source: Front Plant Sci. 2022 Feb 18;12:809069. doi: 10.3389/fpls.2021.809069 (PMC8895299; doi:10.3389/fpls.2021.809069)
Supplement: Supplementary file 2 [file Data_Sheet_2.PDF]

Table S2 Introgression crossing programme between *A. officinalis* x *A. prostratus*

Crosses (No. of chromosome	No of crosses	Berries n (%)	Embryo rescue[1]	Plants established[2] in vitro	in vivo	AV-1 resistant plants[3]
F_1						
A. officinalis (20) x *A.*	80	29 (36.3)	17 (14)	140	104	27 (6)
BC_1						
F_1 AO 234 (30) x *A.*	167	20 (11.9)	4 (4)	55	55	5 (1)
F_1 AO 246 (60) x *A.*	148	0 (0.0)	0	0	0	0
F_1 AO 252 (30) x *A.*	27	8 (29.6)	0	0	0	0
F_1 AO 258 (30) x *A.*	183	22 (12.0)	15 (15)	142	142	57 (9)
F_1 AO 297[4] (30) x *A.*	27	8 (29.6)	0	0	0	0
A. officinalis (20) x F_1 AO	25	0 (0.0)	0	0	0	0
A. officinalis (20) x F_1 AO	48	2 (4.1)	0	0	0	0
BC_2						
BC_1 AO 435 (28) x *A.*	59	10 (16.9)	6(4)	24	20	7 (1)
BC_1 AO 449 (29) x *A.*	75	3 (4.0)	5 (3)	30	22	15 (2)
A. officinalis (20) x BC_1 AO	178	47 (26.4)	31 (22)	193	145	49 (7)
A. officinalis (20) x BC_1 AO	70	14 (20.0)	8 (6)	80	62	36 (2)
A. officinalis (20) x BC_1 AO	6	3 (50.0)	4 (4)	53	40	40 (4)

[1] No. of prepared embryos, bracketed no of established plants;

[2] Embryo based plants were cloned (5-10 plants) and established in vitro and then transferred in vivo (greenhouse);

[3] Bracketed no of crossing occurrence

[4] AO 297 F_1 clone plants segregate in male and female plants
