# Supplementary material for: Transfer of the Dominant Virus Resistance Gene AV-1pro From Asparagus prostratus to Chromosome 2 of Garden Asparagus A. officinalis L
Source: Front Plant Sci. 2022 Feb 18;12:809069. doi: 10.3389/fpls.2021.809069 (PMC8895299; doi:10.3389/fpls.2021.809069)
Supplement: Supplementary file 3 [file Data_Sheet_3.PDF]

**Table S3** Results of the pollen vitality analysis using the FDA test

| No | Generation | Cross parents & Crosses | Chromosome number | AV-1 | Flowers analysed (n) | Pollen counted (n) | Vitality of pollen in % (Mean ± SD) |
|---|---|---|---|---|---|---|---|
| PRO | Pop. | *A. prostratus* | 40 | res | 13 | 10924 | 72.21 ± 10.26 |
| | | *A. officinalis* | | | | | |
| DAR | $F_1$ | cv. Darlise | 20 | susc | 19 | 13915 | 80.12 ± 7.23 |
| GIL | $F_1$ | cv. Gijnlim | 20 | susc | 8 | 3029 | 76.81 ± 8.57 |
| RAV | $F_1$ | cv. Ravel | 20 | susc | 12 | 14669 | 79.52 ± 9.82 |
| BOO | $F_1$ | cv. Boonlim | 20 | susc | 4 | 3098 | 62.72 ± 19.24 |
| SWM | OP | cv. Schwetzinger Meisterschuß | 20 | susc | 9 | 7468 | 76.77 ± 7.65 |
| AO 297 | $F_1$ | BL1 x PRO | 30 | res | 4 | 3283 | 8.19 ± 1.71 |
| AO 250 | $F_1$ | BL1 x PRO | 30 | susc | 4 | 2094 | 7.35 ± 2.14 |
| AO 350 | $BC_1$ | AO 234 x RAV | 28 | res | 4 | 2374 | 3.29 ± 0.99 |
| AO 390 | $BC_1$ | AO 258 x BOO | 29 | res | 14 | 11936 | 54.91 ± 23.45 |
| AO 380 | $BC_1$ | AO 258 x BOO | 27 | res | 10 | 7439 | 45.41 ± 20.29 |
| AO 434 | $BC_1$ | AO 258 x BOO | 23 | res | 4 | 2770 | 32.74 ± 12.14 |
| AO 443 | $BC_1$ | AO 258 x BOO | 29 | res | 10 | 7685 | 36.24 ± 16.08 |
| AO 538 | $BC_2$ | SWM x AO 443 | 20 | res | 16 | 13595 | 80.74 ± 6.27 |
| AO 606 | $BC_2$ | BL2 x AO 390 | 20 | res | 10 | 11385 | 81.88 ± 2.97 |
| AO 618 | $BC_2$ | BL2 x AO 380 | 20 | res | 15 | 13301 | 53.05 ± 16.34 |
| AO 627 | $BC_2$ | BL2 x AO 380 | 20 | susc | 4 | 4495 | 74.74 ± 11.87 |
| AO 632 | $BC_2$ | BL2 x AO 380 | 20 | susc | 4 | 5149 | 82.15 ± 2.95 |
