# Supplementary material for: Transfer of the Dominant Virus Resistance Gene AV-1pro From Asparagus prostratus to Chromosome 2 of Garden Asparagus A. officinalis L
Source: Front Plant Sci. 2022 Feb 18;12:809069. doi: 10.3389/fpls.2021.809069 (PMC8895299; doi:10.3389/fpls.2021.809069)
Supplement: Supplementary file 4 [file Data_Sheet_4.PDF]

Table S4 Samples GBS analysis

Plant	Material	Generation
AO 172-2	*A. officinalis*	BL1
AO 258-5	IH	F_1
Lo9-61	*A. officinalis*	*cv. Boonlim*
AO 140-2	*A. officinalis*	cv. Ravel
AO 390-2	IH	BC_1
AO 4-2	*A. officinalis*	BL4
AO561-1	IH	BC_2
AO 606-1	IH	BC_2
AO 606-2	IH	BC_2
AO 556-5	IH	BC_2
AO 172-1	*A. officinalis*	BL1
AO 589-1	IH	BC_2
AO 570-1	IH	BC_2
AO 553-2	IH	BC_2
AO 553-4	IH	BC_2
AO 380-1	IH	BC_2
AO 597-6	IH	BC_2
AO 584-1	IH	BC_2
AO 607-1	IH	BC_2
AO 380-2	IH	BC_2
AO 610-1	IH	BC_2
AO 610-2	IH	BC_2
AO 555-1	IH	BC_2
AO 3-2	*A. officinalis*	BL3
AO 538-1	IH	BC_2
AO 538-2	IH	BC_2
AO 546-1	IH	BC_2
AO 546-2	IH	BC_2
AO 473-1	IH	BC_1
AO 4-4	*A. officinalis*	BL4
AO 395-10	*A. officinalis*	cv. Ravel
AO 618-1	IH	BC_2
AO 618-2	IH	BC_2
AO 583-4	IH	BC_2
AO 583-5	IH	BC_2
AO 611-3	IH	BC_1
AO 577-1	IH	BC_2
AO 314-8	*A. officinalis*	cv. Schwetzinger Meisterschuß
AO 627-1	IH	BC_2
Lo9-36	*A. officinalis*	cv. Boonlim
AO 147-2	*A. officinalis*	landrace from Usbekistan
AO 503	*A. officinalis*	cv. Fileas F1
AO 342-49	*A. officinalis*	cv. Darlise
AO 488-4	*A. officinalis*	cv. Eposs
AO 404-14	*A. officinalis*	cv. Gijnlim
AO 453-22	*A. officinalis*	cv. Ravel
AO 302-13	*A. officinalis*	cv. Huchels Alpha
AO 303-21	*A. officinalis*	cv. Ariane
AO 630-1	IH	BC_2
AO 602-4	IH	BC_2
AO 617-2	IH	BC_2

Green boxes mark AV-1 resistant plants, IH - Interspecific hybrid
