# Supplementary material for: Transfer of the Dominant Virus Resistance Gene AV-1pro From Asparagus prostratus to Chromosome 2 of Garden Asparagus A. officinalis L
Source: Front Plant Sci. 2022 Feb 18;12:809069. doi: 10.3389/fpls.2021.809069 (PMC8895299; doi:10.3389/fpls.2021.809069)
Supplement: Supplementary file 5 [file Data_Sheet_5.PDF]

Table S5- Samples array and KASP analysis

Plant	Material		Array-Analysis	KASP-Analysis
AO 172-1	*A. officinalis*	BL1	X	
AO 172-2	*A. officinalis*	BL1	X	X
AO 183-3	*A. prostratus*	Wild relative		X
AO 553-4	IH	BC_2		X
AO 514-4	*A. officinalis*	cv. Schwetzinger Meisterschuß		X
AO 709-1	IH	BC_3		X
AO 709-2	IH	BC_3		X
AO 709-4	IH	BC_3		X
AO 709-5	IH	BC_3		X
AO 709-6	IH	BC_3		X
AO 709-7	IH	BC_3		X
AO 709-10	IH	BC_3		X
AO 709-11	IH	BC_3		X
AO 709-12	IH	BC_3		X
AO 709-13	IH	BC_3		X
AO 709-14	IH	BC_3		X
AO 709-15	IH	BC_3		X
AO 709-16	IH	BC_3		X
AO 709-17	IH	BC_3		X
AO 709-18	IH	BC_3		X
AO 709-19	IH	BC_3		X
AO 709-21	IH	BC_3		X
AO 709-22	IH	BC_3		X
AO 709-23	IH	BC_3		X
AO 709-24	IH	BC_3		X
AO 709-25	IH	BC_3		X
AO 709-26	IH	BC_3		X
AO 709-27	IH	BC_3		X
AO 568-8	*A. officinalis*	cv. Schwetzinger Meisterschuß	X	X
AO 538-1	IH	BC_2	X	X
AO 759-1	IH	BC_3		X
AO 759-2	IH	BC_3	X	X
AO 759-3	IH	BC_3	X	
AO 759-4	IH	BC_3	X	X
AO 759-5	IH	BC_3	X	X
AO 759-6	IH	BC_3		X
AO 759-7	IH	BC_3	X	X
AO 759-8	IH	BC_3	X	X
AO 759-9	IH	BC_3	X	X
AO 759-11	IH	BC_3	X	X
AO 759-12	IH	BC_3	X	X
AO 759-13	IH	BC_3	X	X
AO 759-14	IH	BC_3	X	X
AO 759-15	IH	BC_3	X	X
AO 759-16	IH	BC_3	X	X
AO 759-18	IH	BC_3	X	X
AO 759-19	IH	BC_3	X	X
AO 759-20	IH	BC_3	X	X
AO 759-22	IH	BC_3		X
AO 759-23-1	IH	BC_3	X	X
AO 759-23-2	IH	BC_3	X	
AO 759-24	IH	BC_3		X
AO 759-25	IH	BC_3		X
AO 759-26	IH	BC_3		X
AO 759-27	IH	BC_3		X
AO 759-28	IH	BC_3		X
AO 759-29	IH	BC_3		X
AO 759-30	IH	BC_3		X
AO 759-31	IH	BC_3		X
AO 759-32	IH	BC_3		X
AO 759-33	IH	BC_3		X
AO 759-34	IH	BC_3		X
AO 553-1	IH	BC_3	X	X
AO 342-45	*A. officinalis*	cv. Darlise	X	X
AO 779-1	IH	BC_3	X	X
AO 779-2	IH	BC_3		X
AO 779-3	IH	BC_3		X
AO 779-4	IH	BC_3		X
AO 779-5	IH	BC_3		X
AO 779-6	IH	BC_3	X	X
AO 779-7	IH	BC_3		X
AO 779-8	IH	BC_3		X
AO 779-9	IH	BC_3		X
AO 779-10	IH	BC_3	X	X
AO 779-11	IH	BC_3	X	X
AO 779-12	IH	BC_3	X	X
AO 779-13	IH	BC_3	X	X
AO 779-14	IH	BC_3	X	X
AO 779-15	IH	BC_3	X	X
AO 779-16	IH	BC_3	X	X
AO 779-17	IH	BC_3	X	X
AO 779-18	IH	BC_3		X
AO 779-19	IH	BC_3	X	X
AO 779-20	IH	BC_3	X	X
AO 779-21	IH	BC_3		X
AO 779-22	IH	BC_3	X	X
AO 779-23	IH	BC_3		X
AO 779-26	IH	BC_3	X	X
AO 779-27	IH	BC_3		X
AO 779-28	IH	BC_3	X	X
AO 779-29	IH	BC_3		X
AO 779-30	IH	BC_3		X
AO 779-31	IH	BC_3		X
AO 779-32	IH	BC_3		X
AO 779-33	IH	BC_3	X	X
AO 779-35	IH	BC_3	X	X
AO 779-36	IH	BC_3		X
AO 779-37	IH	BC_3	X	X
AO 779-38	IH	BC_3	X	X
AO 779-39	IH	BC_3	X	X
AO 568-9	*A. officinalis*	cv. Schwetzinger Meisterschuß	X	X
AO 835-1	IH	BC_3		X
AO 835-2	IH	BC_3	X	X
AO 835-4	IH	BC_3	X	X
AO 835-5	IH	BC_3	X	
AO 835-8	IH	BC_3	X	
AO 835-9	IH	BC_3	X	
AO 835-10	IH	BC_3		X
AO 835-11	IH	BC_3	X	X
AO 835-12	IH	BC_3	X	X
AO 835-13	IH	BC_3	X	X
AO 835-14	IH	BC_3	X	
AO 835-15	IH	BC_3		X
AO 835-16	IH	BC_3	X	X
AO 835-17	IH	BC_3	X	X
AO 835-18	IH	BC_3	X	X
AO 835-19	IH	BC_3		X
AO 835-20	IH	BC_3	X	X
AO 835-21	IH	BC_3	X	X
AO 835-22	IH	BC_3	X	
AO 835-23	IH	BC_3	X	X
AO 835-24	IH	BC_3	X	X
AO 835-25	IH	BC_3		X
AO 835-27	IH	BC_3	X	X
AO 835-28	IH	BC_3		X
AO 835-30	IH	BC_3		
AO 835-31	IH	BC_3		X
AO 835-32	IH	BC_3		X
AO 835-33	IH	BC_3	X	X
AO 835-34	IH	BC_3		X
AO 835-36	IH	BC_3		X
AO 835-38	IH	BC_3		X
AO 835-39	IH	BC_3		X
AO 835-40	IH	BC_3		X
AO 835-41	IH	BC_3		X
AO 835-42	IH	BC_3	X	
AO 01-2	*A. officinalis*	cv. Pacific 2000		X
AO 140-1	*A. officinalis*	cv. Ravel	X	X
AO 169	*A. officinalis*	cv. Plasenesp	X	X
AO 170-1	*A. officinalis*	cv. Dorsiane	X	X
AO 307-13	*A. officinalis*	cv. Andreas	X	X
AO 325-27	*A. officinalis*	cv. Eposs	X	X
AO 343-8	*A. officinalis*	cv. Schneekopf	X	X
AO 344-3	*A. officinalis*	cv. Primens	X	X
AO 359	*A. officinalis*	cv. Ramada	X	X
AO 404-9	*A. officinalis*	cv. Gijnlim	X	X
AO 586-66	*A. officinalis*	cv. Mondeo	X	X
AO 422	*A. officinalis*	cv. Primaverde	X	X
AO 424-7	*A. officinalis*	cv. Leistungsauslese	X	X
AO 453-22	*A. officinalis*	cv. Thielim	X	X
AO 503	*A. officinalis*	cv. Fileas	X	X
AO 425-9	*A. officinalis*	cv. Ariane	X	X
AO 587	*A. officinalis*	cv. Raffaelo	X	X
AO 650	*A. officinalis*	cv. Spaganiva	X	X
AO 651	*A. officinalis*	Huchels Alpha	X	X
AO 652	*A. officinalis*	cv. Ruhm von Braunschweig	X	X
AO 853-3	*A. officinalis*	Chinese Breeding line 1	X	X
AO 854-1	*A. officinalis*	Chinese Breeding line 2	X	X
AO 855-3	*A. officinalis*	Chinese Breeding line 3	X	X
Lo 9-61	*A. officinalis*	cv. Boonlim	X	X
P 22	*A. officinalis*	cv. Rapsody	X	X

Green boxes mark AV-1 resistant plants, IH - Interspecific hybrid
