# Supplementary material for: Transfer of the Dominant Virus Resistance Gene AV-1pro From Asparagus prostratus to Chromosome 2 of Garden Asparagus A. officinalis L
Source: Front Plant Sci. 2022 Feb 18;12:809069. doi: 10.3389/fpls.2021.809069 (PMC8895299; doi:10.3389/fpls.2021.809069)
Supplement: Supplementary file 6 [file Data_Sheet_6.PDF]

Table S6 Association of marker Ax-553065353 and AV-1 resistance

Material	Plant status	Resistance status	Ax-553065352
A. prostratus parent	Single plant	Resistant	GG
A. officinalis backcross parent	Single plant	Susceptible	CC
AO 538 (BC_2)	Single plant	Resistant	GC
AO 759 (BC_3)	Seed population	10 x susceptible<br>10 x resistant	10 x CC<br>10 x GC
A. officinalis backcross parent	Single plant	Susceptible	CC
AO 553 (BC_2)	Single plant	Resistant	GC
AO 779 (BC_3)	Seed population	10 x susceptible<br>10 x resistant	10 x CC<br>9 x GC; 1 x CC
A. officinalis backcross parent	Single plant	Susceptible	CC
AO 538 (BC_2)	Single plant	Resistant	GC
AO 835 (BC_3)	Seed population	10 x susceptible<br>10 x resistant	10 x CC<br>10 x GC
25 garden asparagus cultivars	Single plants	25 x susceptible	25 x CC
