# Supplementary material for: Transfer of the Dominant Virus Resistance Gene AV-1pro From Asparagus prostratus to Chromosome 2 of Garden Asparagus A. officinalis L
Source: Front Plant Sci. 2022 Feb 18;12:809069. doi: 10.3389/fpls.2021.809069 (PMC8895299; doi:10.3389/fpls.2021.809069)
Supplement: Supplementary file 7 [file Data_Sheet_7.pdf]

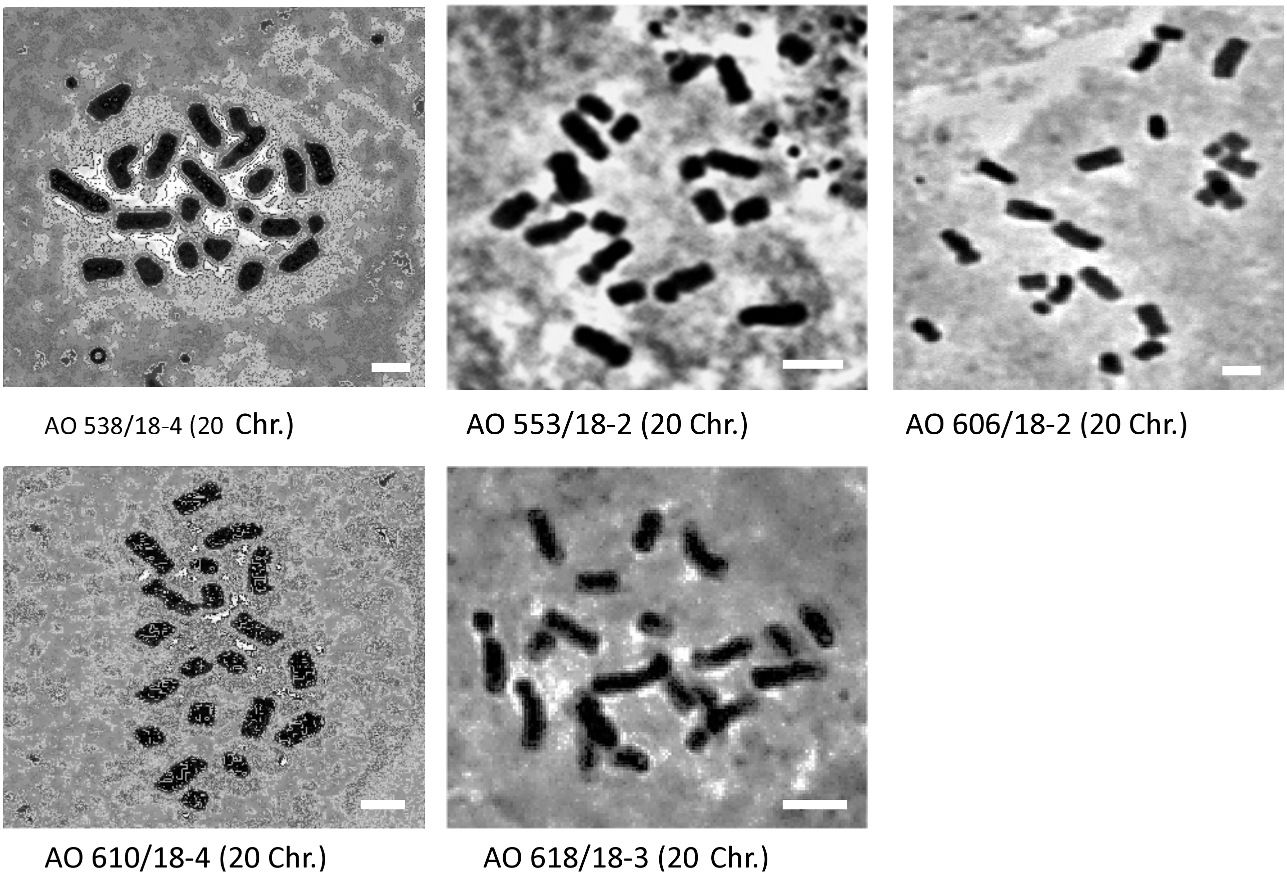

Figure S1 Mitotic metaphase chromosomes in root tip cells of selected BC_2 plants.

Scale bar = 2 µm
