# Supplementary material for: Transfer of the Dominant Virus Resistance Gene AV-1pro From Asparagus prostratus to Chromosome 2 of Garden Asparagus A. officinalis L
Source: Front Plant Sci. 2022 Feb 18;12:809069. doi: 10.3389/fpls.2021.809069 (PMC8895299; doi:10.3389/fpls.2021.809069)
Supplement: Supplementary file 8 [file Data_Sheet_8.PDF]

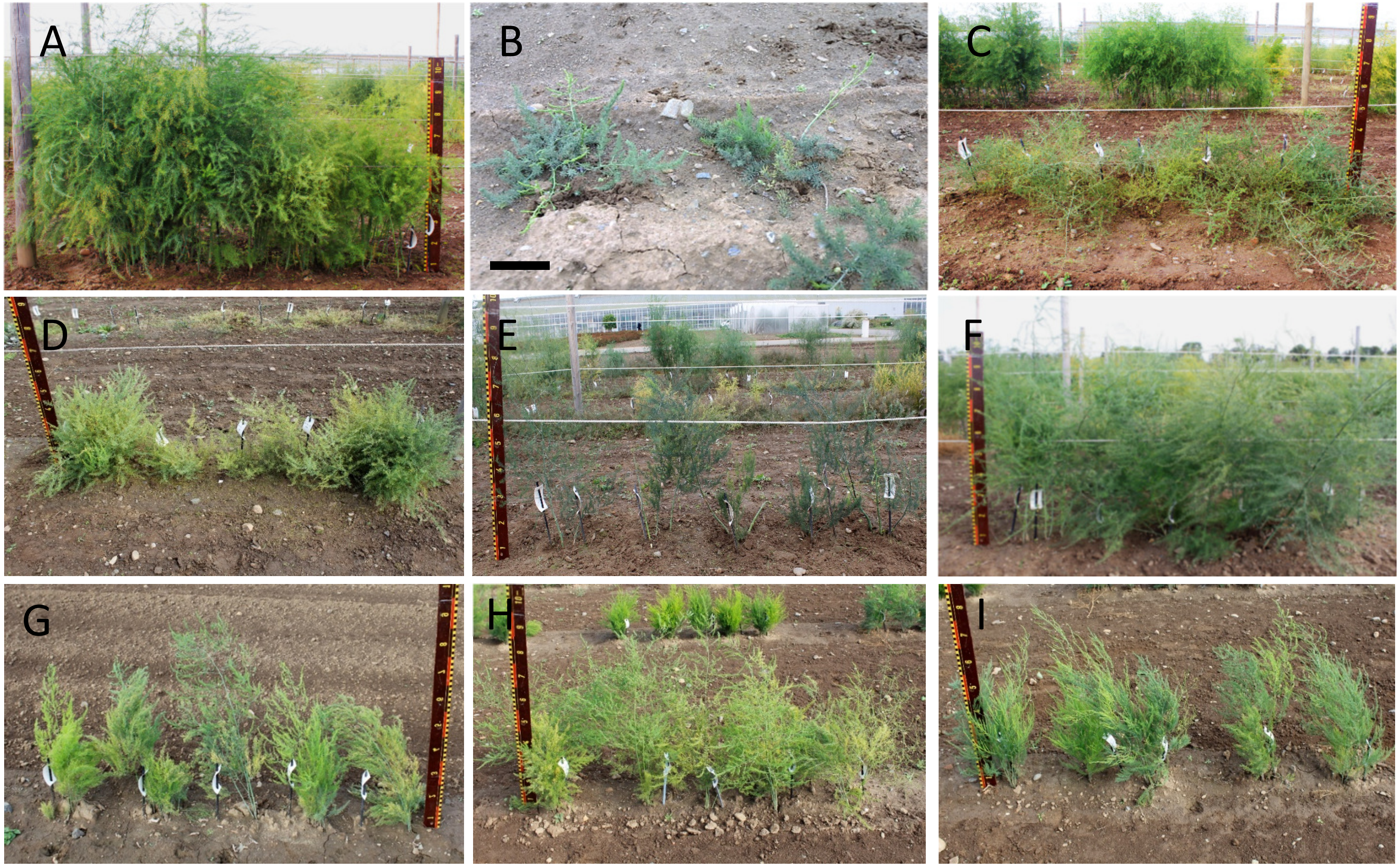

Figure S2 Microplots of asparagus plants in a research field during end of September. Each five one years old plants were replanted in a 1.5 m microplot row. **A** – *A. officinalis* BL1; **B** - Two single plants of *A.prostratus,* the plants died over the second winter (bar = 10 cm); **C** – AO 258 (F_1); **D** - AO 390 (BC_1); **E** – AO 538 (BC_2); **F** – AO 553 (BC_2); **G** – AO 759 (BC_3); **H** – AO 779 (BC_3); **I** – AO 835 (BC_3). Plants **A** and **C-F** are approximately three years old, plants in **G-I** are approximately 1.5 years old and grow 5 month on field. Ruler unit in [cm].
