# Supplementary material for: Transfer of the Dominant Virus Resistance Gene AV-1pro From Asparagus prostratus to Chromosome 2 of Garden Asparagus A. officinalis L
Source: Front Plant Sci. 2022 Feb 18;12:809069. doi: 10.3389/fpls.2021.809069 (PMC8895299; doi:10.3389/fpls.2021.809069)
Supplement: Supplementary file 9 [file Data_Sheet_9.PDF]

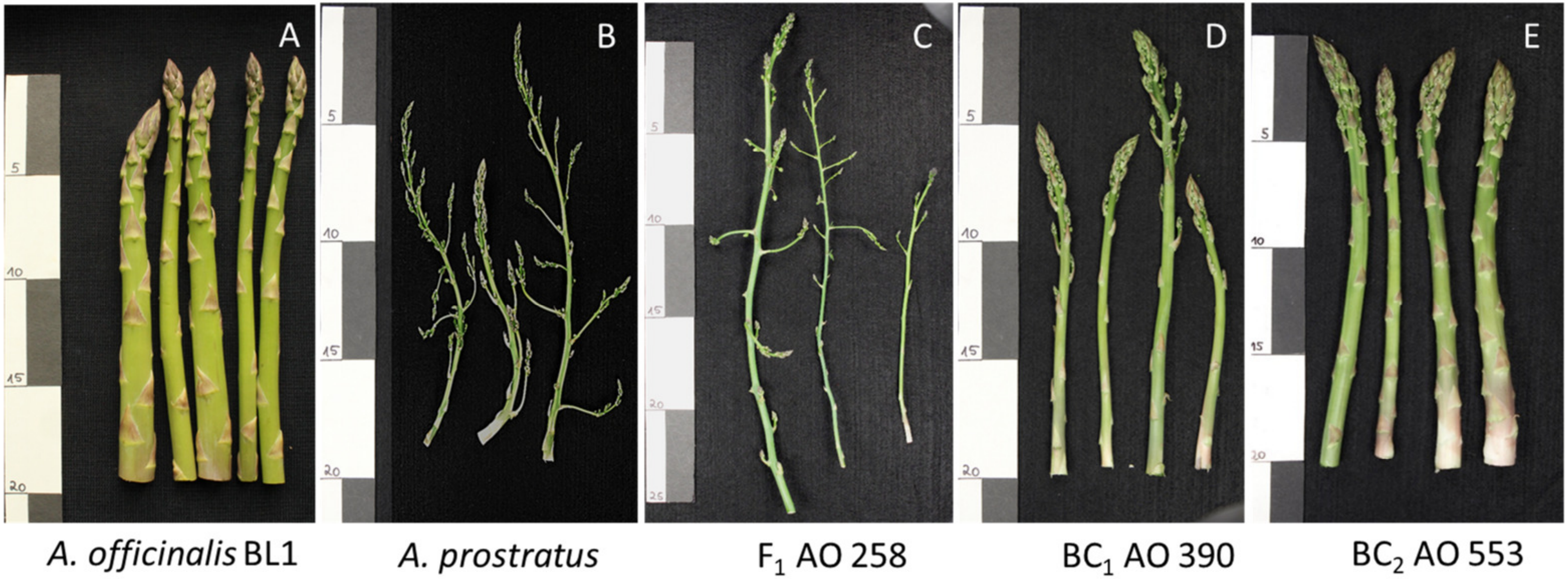

Figure S3 Representative shoot (spear) morphology of the parental plants (A, B) and a single F_1, BC_1 and BC_2 plant (C, D, E), repectively. (Shoots were harvested middle of June. Plant age during harvest time: 3 years thereof two years growing on field. Ruler unit = cm)
