# Supplementary material for: Transfer of the Dominant Virus Resistance Gene AV-1pro From Asparagus prostratus to Chromosome 2 of Garden Asparagus A. officinalis L
Source: Front Plant Sci. 2022 Feb 18;12:809069. doi: 10.3389/fpls.2021.809069 (PMC8895299; doi:10.3389/fpls.2021.809069)
Supplement: Supplementary file 10 [file Data_Sheet_10.PDF]

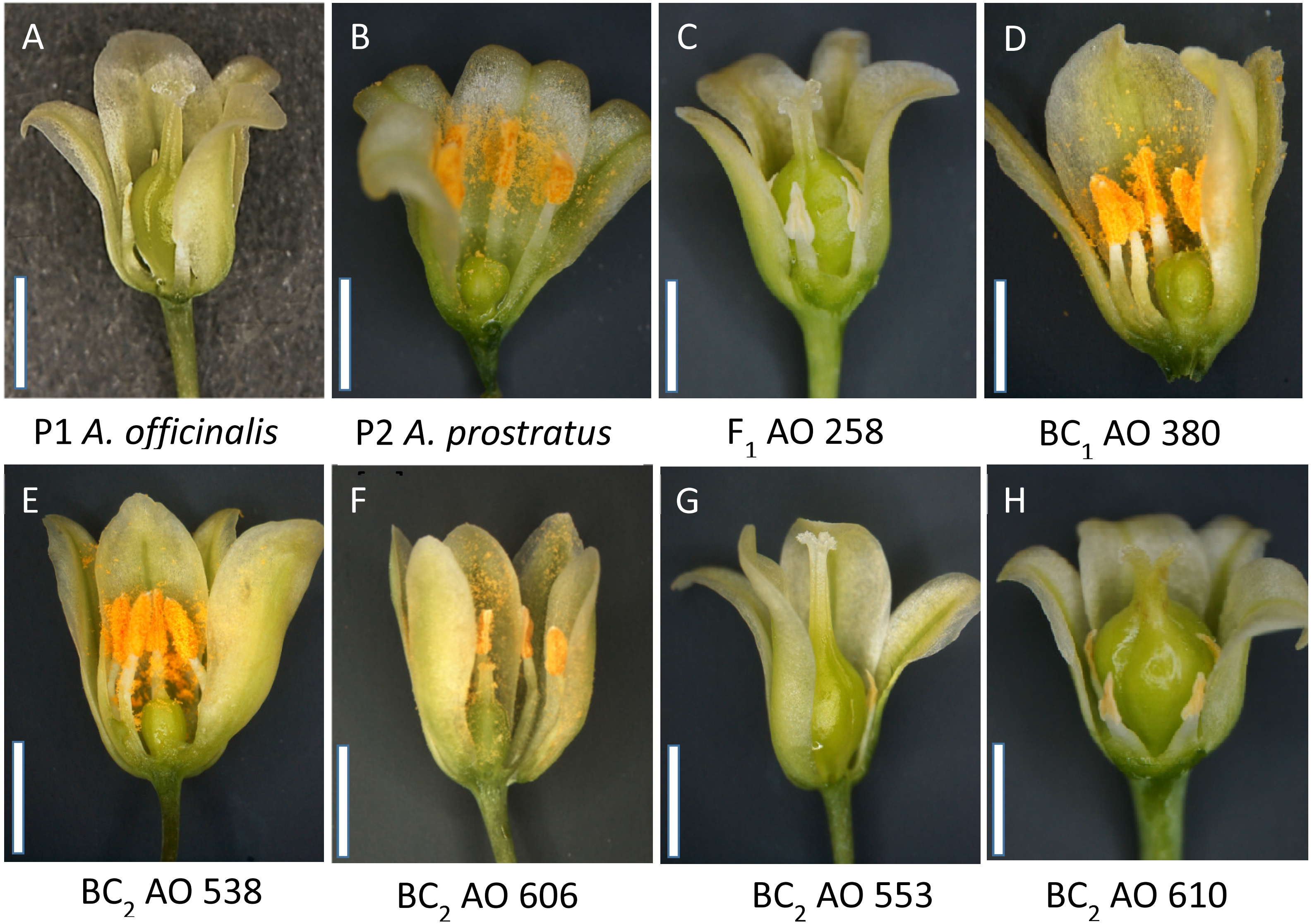

Figure S4 Flower achitecture of the parental plant (A,B), the F_1 generation (c) as well as the backcross progenies BC_1 (D) and BC_2 (E-H), Bar = 5 mm
