# Supplementary material for: Transfer of the Dominant Virus Resistance Gene AV-1pro From Asparagus prostratus to Chromosome 2 of Garden Asparagus A. officinalis L
Source: Front Plant Sci. 2022 Feb 18;12:809069. doi: 10.3389/fpls.2021.809069 (PMC8895299; doi:10.3389/fpls.2021.809069)
Supplement: Supplementary file 11 [file Data_Sheet_11.PDF]

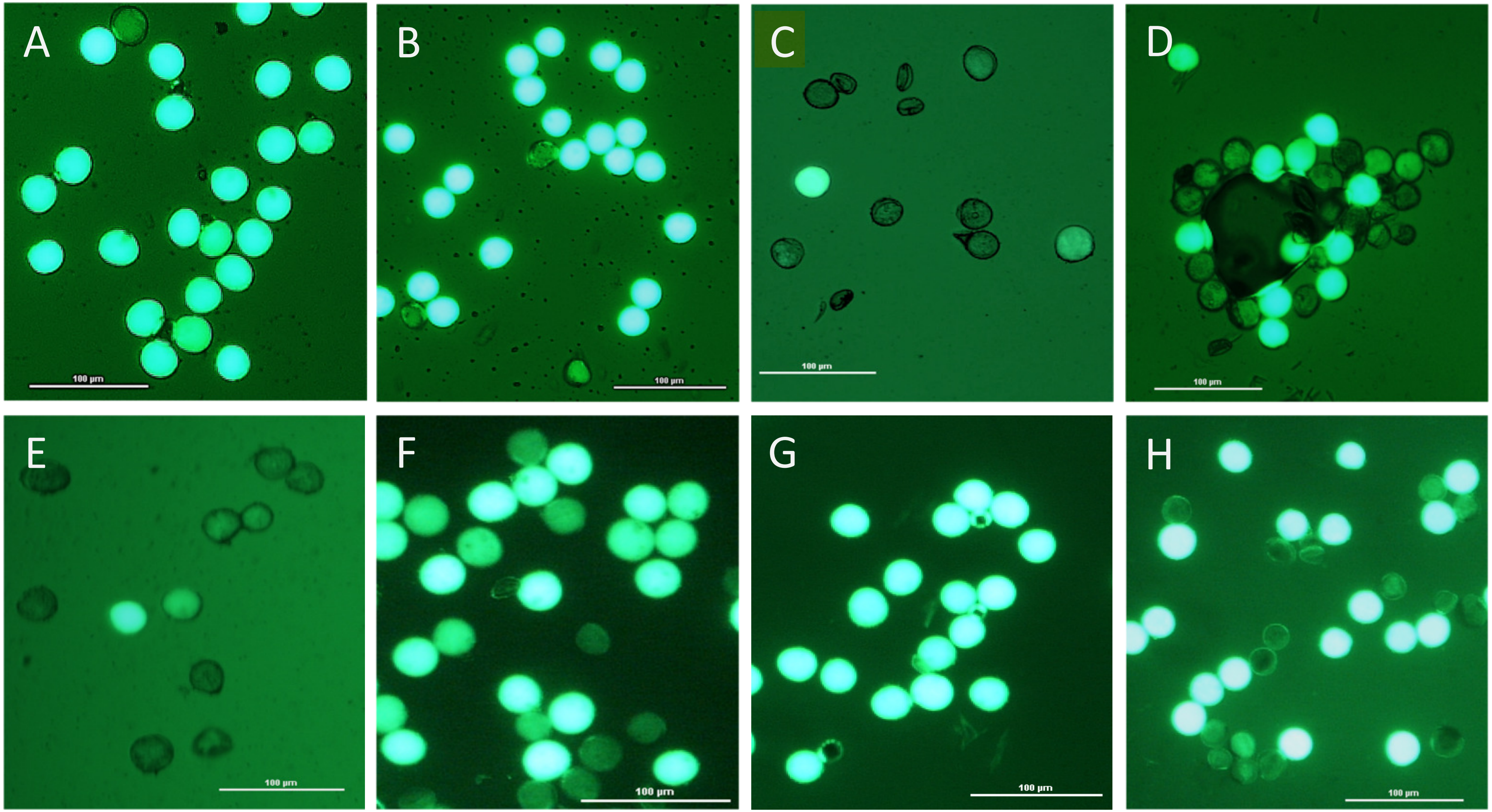

Figure S5 FDA test to assess the pollen vitality. A – *Asparagus prostratus*, B – *A. officinalis*, C - F_1 AO 297, D - BC_1 AO443, E - BC_1 AO380, F – BC_2 AO 606, G – BC_2 AO 635, H – BC_2 AO 618
